# Reconstitution of an active human CENP-E motor

**DOI:** 10.1098/rsob.210389

**Published:** 2022-03-09

**Authors:** Benjamin Craske, Thibault Legal, Julie P. I. Welburn

**Affiliations:** Wellcome Trust Centre for Cell Biology, School of Biological Sciences, University of Edinburgh, Edinburgh, Scotland EH9 3BF, UK

**Keywords:** motor, mitosis, microtubule, motility, CENP-E, kinetochore

## Abstract

CENP-E is a large kinesin motor protein which plays pivotal roles in mitosis by facilitating chromosome capture and alignment, and promoting microtubule flux in the spindle. So far, it has not been possible to obtain active human CENP-E to study its molecular properties. *Xenopus* CENP-E motor has been characterized *in vitro* and is used as a model motor; however, its protein sequence differs significantly from human CENP-E. Here, we characterize human CENP-E motility *in vitro*. Full-length CENP-E exhibits an increase in run length and longer residency times on microtubules when compared to CENP-E motor truncations, indicating that the C-terminal microtubule-binding site enhances the processivity when the full-length motor is active. In contrast with constitutively active human CENP-E truncations, full-length human CENP-E has a reduced microtubule landing rate *in vitro*, suggesting that the non-motor coiled-coil regions self-regulate motor activity. Together, we demonstrate that human CENP-E is a processive motor, providing a useful tool to study the mechanistic basis for how human CENP-E drives chromosome congression and spindle organization during human cell division.

## Graphical abstract

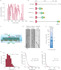

## 1. Introduction

Chromosome alignment and segregation is essential to ensure genomic stability. The mitotic spindle is the physical apparatus that allows the accurate alignment of chromosomes during mitosis. Following the disassembly of the nuclear envelope in prophase, chromosomes are captured by microtubules and aligned in the metaphase plate [1–4]. However, chromosomes at the spindle poles often cannot biorient through this search and capture mechanism, and use a dynein/CENP-E-dependent pathway [5,6]. The microtubule motor protein CENP-E is recruited to the fibrous corona of unattached kinetochores, a large macromolecular structure that maximizes the microtubule-binding surface of kinetochores to favour microtubule capture [2–4,7]. Upon microtubule capture, CENP-E walks towards microtubule plus ends and promotes the lateral to end-on conversion of kinetochores on microtubules [8,9] (reviewed in [10]). CENP-E is recruited to kinetochores through a rapid BubR1-dependent and a slower BubR1-independent pathway [11–13]. Inhibition or depletion of CENP-E in human cells increases the incidence of chromosome misalignments, causes spindle assembly checkpoint activation and results in a prometaphase arrest [14–16], highlighting the essential function of the kinetochore-localized motor during chromosome congression. More recently the kinetochore-bound CENP-E population has been implicated in promoting microtubule flux in prometaphase [17]. CENP-E also localizes to the overlapping microtubules of the spindle midzone and midbody, suggesting roles for CENP-E during the later stages of mitosis [18].

Previous work to reconstitute the activity of native CENP-E fractionated from HeLa cells indicated that the full-length protein was inactive [19]. Thus until now, biochemical characterization studies and *in vitro* reconstitutions of CENP-E activity have used the *Xenopus laevis* CENP-E orthologue. *X. laevis* CENP-E displays processive motility along single microtubules *in vitro* and is required for chromosome alignment in egg extracts [14,20,21]. This has provided important insights into how CENP-E functions at a molecular level. However, human and *X. laevis* CENP-E share only 49% sequence similarity. Divergence is highest within the stalk (human CENP-E aa 341–2054) and tail (human CENP-E aa 2055–2701) regions, which are 46.8% and 44.8% similar to corresponding Xenopus CENP-E residues 342–2214 and 2215–2954, respectively. By contrast, the N-terminal motor domains are relatively well conserved with 85.7% similarity. The human model system is often used for cell biology, functional and structural studies of human kinetochores and cell division. Currently, it is not clear to what extent the large-sequence differences provide properties to human CENP-E distinct from the *Xenopus* CENP-E orthologue to mediate chromosome segregation in humans. In this study, we report that both truncated and full-length human CENP-E motors are capable of processive motility. We find truncated CENP-E is constitutively active and processive *in vitro*, capable of unidirectional movement along microtubules. By contrast, only a fraction of full-length human CENP-E motors are active, yet more processive than truncated CENP-E upon a successful collision with the microtubule. This indicates that the long non-motor region interferes with the motile properties of full-length CENP-E *in vitro*. Overall, the reconstitution of active human CENP-E motors obtained in this study represents a useful resource for the study of the mechanistic basis for chromosome segregation in humans.

## 2. Results

### 2.1. Truncated human CENP-E constructs are motile and processive

**Figure 1.**
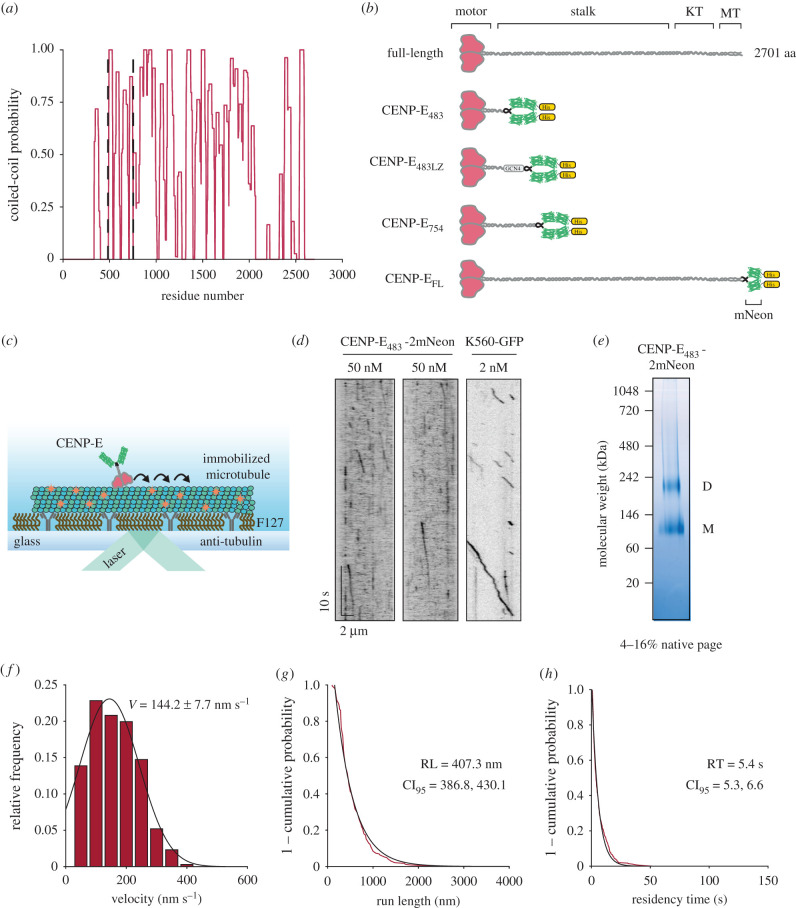
The first predicted coiled-coil of human CENP-E weakly facilitates dimerization of motor domains. (*a*) Coiled-coil prediction of full-length CENP-E by Paircoil2. Dashed vertical lines represent truncations. (*b*) Constructs used in this study. KT = kinetochore-binding domain, MT = second microtubule-binding site, GCN4 = GCN4 leucine zipper domain, His = hexahistidine tag, mNeon = mNeonGreen fluorescent protein. (*c*) Schematic representation of a single-molecule motility assay. (*d*) Kymographs of CENP-E_483_-2mNeon and K560-GFP at indicated nanomolar concentrations for motility assays. (*e*) Native PAGE analysis of purified CENP-E_483_-2mNeon oligomeric status. M = monomer, D = dimer. (*f*) Histogram representation of velocities for CENP-E_483_-2mNeon (*n* = 346) at 50 nM fit to a single Gaussian distribution (*r*^2^ = 0.978), mean of the Gaussian fit ± s.e.m. are reported in the graph, median ± s.e = 131.6 ± 4.1 nm s^−1^. (*g*) 1 − cumulative frequency distribution of run lengths for CENP-E_483_-2mNeon at 50 nM (*n* = 346) fit to a single-exponential decay (*r*^2^ = 0.982). (*h*) 1 − cumulative frequency distribution of residency times for CENP-E_483_-2mNeon at 50 nM (*n* = 346) fit to a single-exponential decay (*r*^2^ = 0.990).

As human full-length CENP-E has been shown to be inactive [19], we first tested whether a minimal human CENP-E motor displayed any motility. We designed several N-terminal truncations containing the motor domain. Human CENP-E is predicted to contain over 20 discontinuous coiled-coils within its stalk and C terminus (figure 1*a,b*). The first putative coiled-coil of human CENP-E is predicted to form between residues 334–401 (figure 1*a*) by PairCoil2 [22]. A minimal truncated *Xenopus* CENP-E_1-473_ construct containing the motor domain, a single coiled-coil between residues 335–392 and terminating at Thr-473 with a C-terminal GFP tag, is processive *in vitro* [20]. We therefore designed a similar construct of human CENP-E, which we refer to as CENP-E_483_-2mNeon, followed by two tandem mNeonGreen fluorophores for recombinant expression and purification from insect cells (figure 1*b*; electronic supplementary material, figure S1B).

Next, we tested whether human CENP-E_483_-2mNeon walks processively on microtubules using *in vitro* reconstitution and single-molecule imaging with total internal reflection fluorescence (TIRF) microscopy. Processive landing events of human CENP-E_483_-2mNeon on immobilized microtubules were rare at the low nanomolar concentrations required for single-molecule imaging (figure 1*c,d*; electronic supplementary material, figure S1C), in contrast with constitutively active Kinesin-1 (K560-GFP) (figure 1*d*). The majority of CENP-E_483_-2mNeon microtubule-binding events were short-lived interactions, with only a small fraction undergoing continuous unidirectional movement along microtubules (figure 1*d*). This suggested to us the motor may not be stable, despite eluting as a single peak by size-exclusion chromatography (electronic supplementary material, figure S1A). To test whether CENP-E_483_-2mNeon was a stable dimer, we analysed the oligomeric status of purified CENP-E_483_-2mNeon by native PAGE. We detected the presence of two separate protein species migrating at approximately 110 kDa and approximately 230 kDa (figure 1*e*), while under denaturing conditions, CENP-E_483_-2mNeon runs at approximately 110 kDa (electronic supplementary material, figure S1B). Given that the predicted monomeric molecular weight of CENP-E_483_-2mNeon is 109 kDa, this result indicates that purified CENP-E_483_-2mNeon exists dynamically as a mixture of monomers and dimers in solution (figure 1*e*). Thus, the first coiled-coil within the stalk of human CENP-E supports only weak dimerization of the motor, as previously reported for *Xenopus* CENP-E [21].

We next measured the behaviour of CENP-E_483_-2mNeon motors *in vitro* at 50 nM, to increase the probability of detecting processive events. We found that human CENP-E_483_-2mNeon motors exhibited an average velocity of 144.2 ± 7.7 nm s^−1^ when moving unidirectionally on the microtubule (figure 1*f*). This velocity was approximately 10-fold faster than a previously reported gliding speed for a truncated human CENP-E construct [23]. With a run length of 407.3 nm (95% confidence interval, CI_95_ [386.9, 430.1] nm), we found that CENP-E_483_-2mNeon motors exhibited a relatively long residency time of 5.41 s (95% confidence interval, CI_95_ [5.29, 6.56] s) on the microtubule lattice (figure 1*g,h*). Kymograph analysis indicated that human CENP-E_483_-2mNeon often exhibited discontinuous motion and frequently paused during processive runs, with recorded velocities ranging from 16.4 nm s^−1^ up to 388.9 nm s^−1^ (figure 1*f*). Similarly, heterogeneity has also been previously reported in the motility of truncated *Xenopus* CENP-E_1-473_ motors *in vitro* [14,20].

### 2.2. Reconstitution of robust processive motility by human CENP-E through stabilization of its dimeric stalk

**Figure 2.**
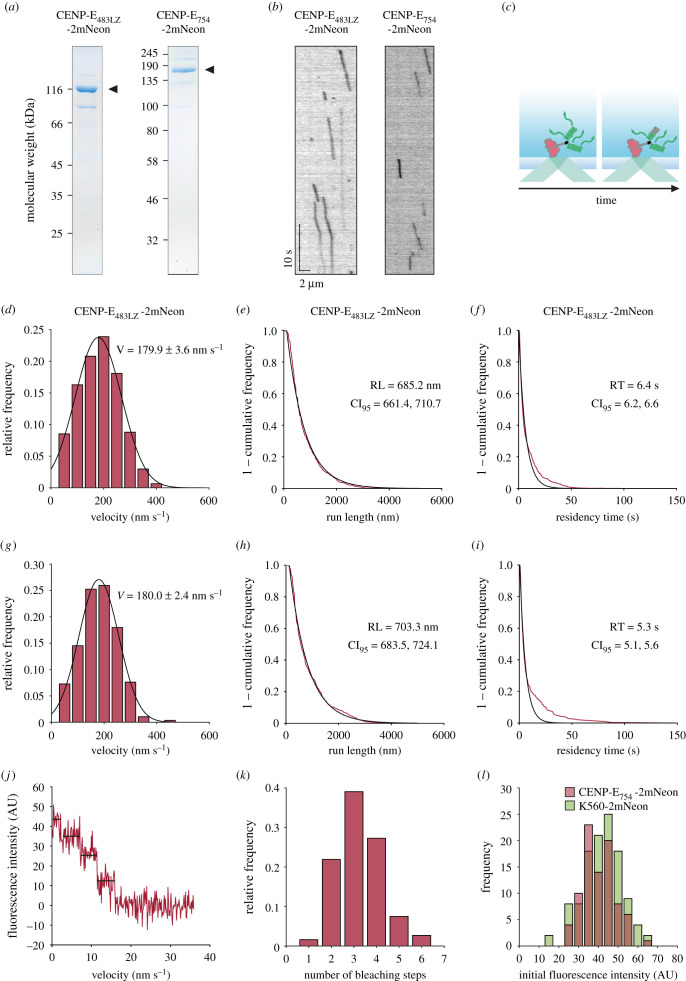
(*Opposite*.) Stable CENP-E dimers are robustly processive *in vitro*. (*a*) Coomassie stained gel of purified CENP-E_483LZ_-2mNeon and CENP-E_754_-2mNeon after SDS-PAGE. Arrowheads indicate purified protein. (*b*) Kymographs of 5 nM CENP-E_483LZ_-2mNeon and 3.5 nM CENP-E_754_-2mNeon moving along single microtubules. (*c*) Schematic representation of photobleaching and intensity analysis assay. (*d*) Histogram distribution of CENP-E_483LZ_-2mNeon velocities (*n* = 774) fitted to a single Gaussian distribution (*r*^2^ = 0.992), mean of the Gaussian fit ± s.e.m. are reported in the graph, median ± s.e. = 160.3 ± 2.7 nm s^−1^. (*e*) 1 − cumulative frequency of run lengths measured for CENP-E_483LZ_-2mNeon (*n* = 774) and fitted to a single-exponential distribution (*r*^2^ = 0.986). (*f*) 1 − cumulative frequency of residency times measured for CENP-E_483LZ_-2mNeon (*n* = 774) and fit to a single-exponential distribution (*r*^2^ = 0.969). (*g*) Histogram distribution of CENP-E_754_-2mNeon velocities (*n* = 289) fit to a single Gaussian distribution (*r*^2^ = 0.996), mean of the Gaussian fit ± s.e.m. are reported in the graph, median ± s.e. = 154.3 ± 4.0 nm s^−1^. (*h*) 1 − cumulative frequency of run lengths measured for CENP-E_754_-2mNeon (*n* = 289) and fit to a single-exponential distribution (*r*^2^ = 0.993). (*i*) 1 − cumulative frequency of residency times measured for CENP-E_754_ (*n* = 289) and fit to a single-exponential distribution (*r*^2^ = 0.966). (*j*) Example four-step photobleaching trace of CENP-E_754_-2mNeon. (*k*) Histogram distribution of CENP-E_754_-2mNeon bleaching steps (*n* = 187). (*l*) Initial fluorescence intensity distribution of CENP-E_754_-2mNeon (*n* = 88) and K560-2mNeon (*n* = 117).

To stabilize the CENP-E motor as a dimer, we artificially dimerized truncated human CENP-E construct by fusing a GCN4 leucine zipper domain to the C terminus of CENP-E_483_-2mNeon and purified CENP-E_483LZ_-2mNeon (figures 1*b* and 2*a*). This approach has been successful in stabilizing the dimeric state of human KIF1A truncations and reconstituting the superprocessive motility of KIF1A *in vitro* [24]. Single molecules of CENP-E_483LZ_-2mNeon walked along microtubules with an average velocity of 179.9 ± 3.6 nm s^−1^, similar to that measured for CENP-E_483_-2mNeon (figure 2*b*–*d*; electronic supplementary material, movie S1). However, CENP-E_483LZ_-2mNeon motors were more processive than the weakly dimeric CENP-E_483_-2mNeon, displaying a run length of 685.2 nm (95% confidence interval, CI_95_ [661.4, 710.7] nm) and a maximum recorded run of 4.4 µm (figure 2*b*–*e*). CENP-E_483LZ_-2mNeon motors demonstrated residency times of 6.36 s (95% confidence interval, CI_95_ [6.17, 6.56] s) on the microtubule (figure 2*f*). Similarly to CENP-E_483_-2mNeon, we found that processive runs of CENP-E_483LZ_-2mNeon were discontinuous but often included pauses mid-run, leading to longer total residency times on the microtubule before detachment (figure 2*b,f*)

As full-length human CENP-E is a homodimer in solution [19], we hypothesized that the dimerization was stabilized by the multiple coiled-coils within the native stalk region. In line with this, subsequent coiled-coils scored higher in Paircoil2 probabilities than the first coiled-coil 334–401 (figure 1*a*). This was confirmed by Alphafold2/ColabFold which predicts residues 345–399 to be coiled-coils (electronic supplementary material, figure S2A–E), although it does not predict the dimerization region with confidence [25,26]. The third highest ranked model was the most reminiscent of a typical kinesin homodimer; however, this model contained breaks and steric clashes within the predicted coiled-coil region (electronic supplementary material, figure S2C). We next generated a truncated CENP-E_754_-2mNeon containing five predicted coiled-coils present in the native stalk of CENP-E. We found that CENP-E_754_-2mNeon was processive, with an average speed of 180.0 ± 2.4 nm s^−1^ (figure 2*g*; electronic supplementary material, movie S2), similar to CENP-E_483LZ_-2mNeon and CENP-E_483_-2mNeon (figures 1*f* and 2*d*). Thus, the coiled-coils in the stalk of CENP-E stabilize homodimerization of the motor domains and facilitate processivity. Single-molecule analysis of CENP-E_754_-2mNeon on microtubules revealed a run length of 703.3 nm (95% confidence interval, CI_95_ [683.53, 724.11] nm) and a residency time of 5.3 s (95% confidence interval, CI_95_ [5.07, 5.56] s) (figure 2*d*–*i*). Photobleaching assays and intensity analysis indicated that CENP-E_754_-2mNeon motors typically bleached in three or four steps and their initial intensities were similar to a purified K560 construct fused to two tandem mNeonGreen tags, referred to as K560-2mNeon (figure 2*c*,*j*–*l*). 51.3% of the CENP-E_754_-2mNeon motors from the purified fraction were motile. This indicates that human CENP-E motor is active and processive in the absence of the coiled-coil C-terminal domain.

### 2.3. Full-length human CENP-E is predominantly inactive but becomes processive upon microtubule binding

**Figure 3.**
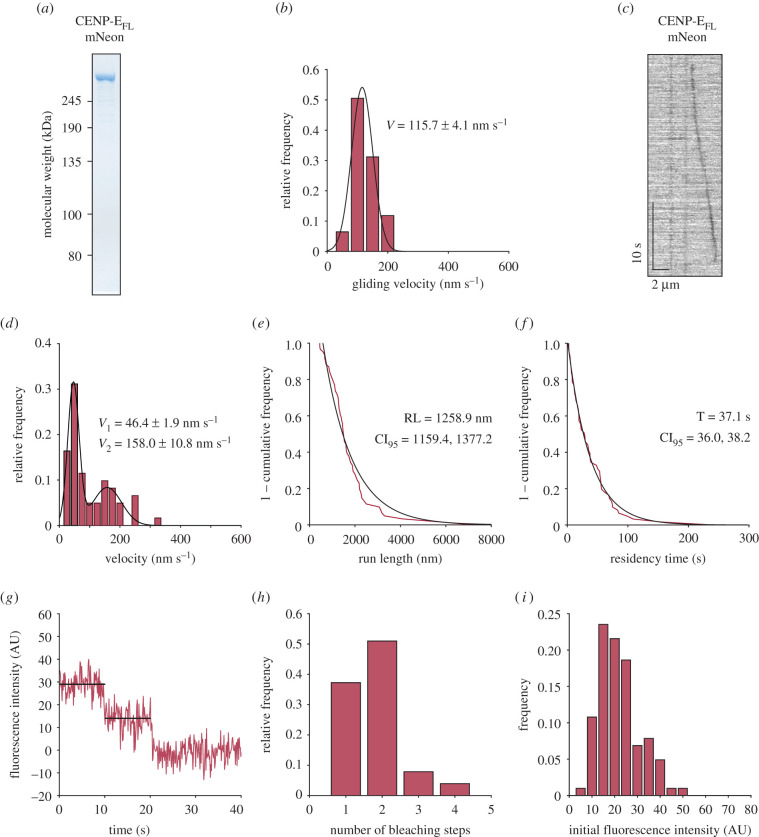
Full-length human CENP-E is a processive motor. (*a*) Coomassie stained gel of purified CENP-E_FL_-mNeon after SDS-PAGE. (*b*) Histogram distribution for microtubule gliding velocities of CENP-E_FL_-mNeon (*n* = 93), mean of the Gaussian fit ± s.e.m. are reported. (*c*) Example of a kymograph showing a single-CENP-E_FL_-mNeon dimer moving along a microtubule. CENP-E_FL_-mNeon was imaged at 12.5 nM. (*d*) Histogram distribution of CENP-E_FL_-mNeon velocities (*n* = 61) fitted to a double Gaussian distribution (*r*^2^ = 0.958), mean of the Gaussian fit ± s.e.m. are reported. (*e*) 1 − cumulative frequency of run lengths measured for CENP-E_FL_-mNeon (*n* = 61) and fitted to a single-exponential distribution (*r*^2^ = 0.951). (*f*) 1 − cumulative frequency of residency times measured for CENP-E_FL_-mNeon (*n* = 61) and fitted to a single-exponential distribution (*r*^2^ = 0.995). (*g*) Example two-step photobleaching trace of CENP-E_FL_-mNeon. (*h*) Histogram distribution of CENP-E_FL_-mNeon bleaching steps (*n* = 102). (*i*) Initial fluorescence intensity distribution of CENP-E_FL_-mNeon (*n* = 102).

Previous work reported that the full-length human CENP-E motor purified from human cells is inactive *in vitro* [19]. We expressed and purified full-length human 692 kDa CENP-E-mNeonGreen, referred to as CENP-E_FL_-mNeon, from insect cells (figure 3*a*). We next carried out microtubule gliding assays by tethering CENP-E to the coverslip and flowing in free microtubules in solution with ATP, to assess whether CENP-E_FL_-mNeon is active. Many microtubules did not glide along the coverslip, despite binding to immobilized full-length motors. Notably, some microtubules bound and pivoted on the surface (electronic supplementary material, movie S3), as previously published [19]. A subset of microtubules glided along the coverslip surface, with an average velocity of 115.7 nm s^−1^ (figure 3*b*), in agreement with our single-molecule velocities for truncated human CENP-E motors.

**Figure 4.**
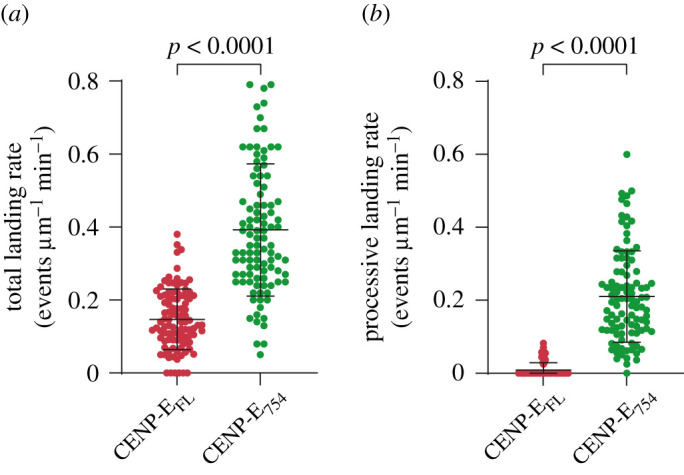
The non-motor regions of human CENP-E regulate processive motility. (*a*) Quantification of total landing rates for CENP-E_FL_-mNeon (*n* = 98, *n* = number of microtubules) and CENP-E_754_-2mNeon (*n* = 100, *n* = number of microtubules). Welch's *t*-test, *p* < 0.0001. (*b*) Quantification of processive landing rates for CENP-E_FL_-mNeon (*n* = 98, *n* = number of microtubules) and CENP-E_754_-2mNeon (*n* = 100, *n* = number of microtubules). Welch's *t*-test, *p* < 0.0001.

We next tested whether single-CENP-E_FL_-mNeon motors displayed any motility on microtubules *in vitro*, using 12.5 nM in our reconstitution assays. We found that CENP-E_FL_-mNeon motors predominantly bound to the lattice in a static manner. However, we observed some single molecules moving processively along the microtubule (figure 3*c*–*i*; electronic supplementary material, movie S4), representing 6.1% of the purified CENP-E_FL_-mNeon motors which bound to microtubules. In contrast with CENP-E_754_-2mNeon, CENP-E_FL_-mNeon run length increased 1.8-fold with a run length of 1258.9 nm (95% confidence interval, CI_95_ [1159.42, 1377.22] nm) and a sevenfold increase in residency time to 37.1 s (95% confidence interval, CI_95_ [36.04, 38.15] s) (figure 3*e*,*f*). We frequently observed discontinuity in CENP-E_FL_-mNeon motion on the microtubule and variation in the recorded velocities (figure 3*d*). CENP-E_FL_-mNeon displayed a bimodal distribution of velocities and the histogram data were fitted to two overlapping Gaussians (figure 3*d*). The majority of motile CENP-E_FL_-mNeon molecules were slow-moving population of motors, moving at 46.4 ± 1.88 nm s^−1^ and would often exhibit paused phases during a single-processive run (figure 3*d*). The slow population of CENP-E_FL_-mNeon was a mixture of full-length molecules moving at slower continuous speeds, as well as motors moving at velocities greater than 150 nm s^−1^ but exhibiting intermittent pauses between periods of unidirectional movement. Yet, a distinct population of CENP-E_FL_-mNeon motors were fast-moving at an average velocity of 157.98 ± 10.77 nm s^−1^, similar to the constitutively active-truncated CENP-E constructs characterized above (figure 2*d*,*g*). The heterogeneity of the motor behaviour is seen in the distribution of velocities (figure 3*d*). We also found that full-length CENP-E landed on the lattice much less frequently than truncated motors (figure 4*a,b*). CENP-E_FL_-mNeon had a landing rate of 0.147 ± 0.008 events µm^−1^ min^−1^, whereas CENP-E_754_-2mNeon had a higher landing rate of 0.392 ± 0.008 events µm^−1^ min^−1^ (figure 4*a*). Importantly, the processive landing rate of CENP-E_FL_-mNeon was 0.009 ± 0.002 events µm^−1^ min^−1^, which was approximately 20-fold lower than the 0.210 ± 0.012 events µm^−1^ min^−1^ observed for truncated CENP-E_754_-2mNeon motors (figure 4*b*). Thus, our *in vitro* reconstitution experiments indicate that a large fraction of purified full-length CENP-E molecules are not active in our assay conditions. However, purified full-length human CENP-E molecules that are active are highly processive upon a successful collision with the microtubule.

## 3. Discussion

Taken together, we show that the human CENP-E motor is an active and processive plus-end directed motor. The majority of full-length CENP-E motors move at a slow average velocity of 46.4 ± 1.88 nm s^−1^, with a fraction moving at a comparable velocity to constitutively active-truncated motors (figures 2*d*,*g* and 3*d*). Similar behaviour has been previously reported for kinesin-1, whereby motile full-length KIF5B molecules exhibit discontinuity in their processive motion and display a much slower velocity than the KIF5B tail-truncated mutant [27]. Processive full-length CENP-E motors exhibited higher run lengths and residency times than truncated CENP-E motors. Here, we show this is due to the increased stabilization of the dimer through the extensive coiled-coils when compared to truncated CENP-E_483_-2mNeon. The increase in processivity may also be due to the presence of a non-motor microtubule-binding site at the far C terminus of full-length CENP-E, as CENP-E_754_-2mNeon lacks this region [28–30]. Many kinesins have a second non-specific microtubule-binding site, which increases their residency time and processivity [31–33].

Our observation that full-length CENP-E activity is relatively variable may explain why previous attempts to reconstitute microtubule gliding activity of HeLa extract purified CENP-E were negative [19]. Misfolding of the C terminus of CENP-E during expression could also occur, although we believe this is unlikely because CENP-E purified from human cells is not active [19]. We favour the idea that the coiled-coils of full-length CENP-E may increase the conformational entropy of the motor *in vitro* and interfere with microtubule binding and processivity, leading to heterogeneity of our motor population. *In vitro* reconstitutions with full-length *Xenopus* CENP-E indicate that the fraction of active motor is increased when coupled to a bead *in vitro*, indicating a potential mechanism where engagement of the C terminus interacting with a cargo (i.e. the bead in that study) promotes CENP-E motor activity [14]. We propose interacting partners at the outer corona or the kinetochores could reorganize the coiled-coils regions, stabilize an active conformation of CENP-E and coordinate its processive transport activity similarly to activation of other kinesin motors [34–38]. Several proteins have been described to interact with CENP-E at kinetochores including BubR1, CLASP1/2, PP1 and CENP-F [11,39–42]. Super-resolution imaging of kinetochores indicates that CENP-E has a compact conformation at the outer corona and kinetochores, close to Ndc80, CENP-F and Spindly [43,44]. *In vitro*, full-length *Xenopus* CENP-E under load stalls at an average force of 4.6 pN but surprisingly maintains a short length of 45 nm when transporting beads under the application of a sidewards force [45]. Thus, activated CENP-E may maintain a compact conformation during transport of heavy-load cargoes, which includes pulling of chromosomes towards the equator [46] and potentially sliding cross-linked microtubules of the spindle [17,47].

Overall human CENP-E appears to be a less active motor than the *Xenopus* orthologue of CENP-E. Here, we show that truncated human CENP-E has an average velocity of 180.0 ± 2.4 nm s^−1^ and a typical run length of 703.3 nm. Truncated *Xenopus* CENP-E_473_ was first reported as a slow motor with an average speed of 8 nm s^−1^ [20]. However, subsequent reconstitutions with truncated *Xenopus* CENP-E_473_ and full-length *Xenopus* CENP-E demonstrated 50-fold higher velocities of approximately 300 nm s^−1^ and 400 nm s^−1^ respectively, and average run lengths of 1.5–2.5 µm [14,48]. The presence of the C-terminal microtubule-binding site in full-length *Xenopus* CENP-E was not reported to enhance CENP-E processivity, in contrast with what we observe for human CENP-E (figures 2*d,g* and 3*d*) [14]. These discrepancies could be attributed to species divergence. For example, human and *Xenopus* CENP-E proteins share only 49% sequence similarity across their entire length. *Xenopus laevis* CENP-E is 253 residues longer than human CENP-E, with a large insertion C-terminal to the kinetochore-targeting domain. It is also likely we are missing regulatory partners that would stabilize the coiled-coil and kinetochore-binding region of CENP-E to optimize motor activity. Through its C terminus, CENP-E binds a number of partners such as BubR1 and other proteins at the outer corona, whose identity is currently not known [2,11,13]. This is an outstanding question in the field. Recent studies have highlighted previously unappreciated localization patterns of human CENP-E at overlapping microtubule bundles and to the detachable fibrous corona in human cells [2,4,17]. Whether *Xenopus* CENP-E is also recruited to these subcellular regions, or whether this is a human-specific CENP-E function, is not currently known. Given that CENP-E interacts with multiple partners in distinct locations, it will be important to define how the regulatory partners regulate CENP-E structure and function, and how they can affect the load-bearing capacities of CENP-E to fulfil its mitotic functions.

## 4. Experimental procedures

### 4.1. Protein expression and purification

The sequences for the CENP-E-mNeonGreen gene were made synthetically for this study and are deposited on addgene. mNeonGreen gene was synthesized by Genewiz. Three synthetic DNA fragments of human CENP-E, codon optimized for insect cell expression, were ordered from Gen9. Each DNA fragment contained 100 bp of overlapping fragments and was amplified by PCR and purified. DNA was transformed into competent BY4741 *Saccharomyces cerevisiae* as described in [49] using an equimolar ratio of each three fragments and pRS415 vector, previously linearized with SmaI. Briefly, PEG, lithium acetate and herring sperm DNA were incubated with the DNA to be assembled and added to 50 µl of competent cells. After a 30 min incubation at 30°C, DMSO was added and the cells were heat shocked at 42°C. The cells were then spun down, re-suspended in 400 µl of 5 mM CaCl_2_ and plated on synthetic defined medium without leucine. Genes encoding full-length *Homo sapiens* CENP-E were amplified by PCR and inserted into a pFastBac1 vector backbone, with a 3C PreScission Protease cleavage site, mNeonGreen fusion protein and a hexahistidine tag located at the C terminus. Truncated *Homo sapiens* CENP-E constructs were generated by PCR amplification of the codon optimized CENP-E sequence as a template. PCR products were digested ligated into a pFastBac1 vector containing 2 x tandem mNeonGreen fusion proteins and a hexahistidine tag at the C terminus. K560-2mNeon was generated by PCR amplifying the *Homo sapiens* KIF5B sequence (amino acids 1–560) and inserting into a pET3aTR vector [50] containing 2 x tandem mNeonGreen fusion proteins and a hexahistidine tag at the C terminus.

Recombinant human CENP-E proteins were expressed using the baculovirus system in Sf9 cells. Cells were harvested 48–62 h after infection and stored at −70°C until use. Harvested cells were resuspended in CENP-E lysis buffer (50 mM HEPES pH 7, 300 mM KCl, 40 mM imidazole, 1 mM MgCl_2_, 1 mM EGTA, 0.1 mM ATP and 5 mM beta-mercaptoethanol) supplemented with 1 mM PMSF, 5 µg ml^−1^ DNase and 1 x cOmplete protease inhibitor tablet per 50 ml. Cells were lysed in a dounce homogenizer with 30–40 strokes. The lysate was cleared by centrifugation at 40 000 rpm in a Type 45 Ti rotor for 60 min at 4°C and applied onto a pre-equilibrated HisTrap HP column (GE Healthcare) in CENP-E lysis buffer at 4°C. HisTrap columns were washed with 40 column volumes of CENP-E lysis buffer. Proteins were eluted with 250 mM imidazole. Elution fractions were concentrated, centrifuged at 13 300 rpm for 15 mins at 4°C and then loaded onto a Superose 6 Increase 10/300 column (GE Healthcare) pre-equilibrated with CENP-E gel filtration buffer (50 mM HEPES pH 7, 300 mM KCl, 1 mM MgCl_2_, 1 mM EGTA, 0.1 mM ATP and 1 mM DTT). Fresh CENP-E proteins were used for all *in vitro* motility assays due to deterioration in activity after freezing.

*Homo sapiens* K560-GFP was purified using a previously described protocol [51] omitting the final microtubule bind and release step, snap frozen and stored at −70°C. K560-2mNeon was transformed in *E. coli* BL21 CodonPlus (DE3) RIL (Agilent Technologies). Transformed BL21 cells were grown to OD_600_ = 0.6 then cooled to 20°C before induction with 0.5 mM IPTG for 3–4 h at 20°C. Frozen pellets were resuspended in K560 lysis buffer (50 mM Tris pH 7.5, 300 mM KCl, 40 mM imidazole, 1 mM MgCl_2_, 1 mM EGTA, 0.1 mM ATP and 5 mM beta-mercaptoethanol) supplemented with 1 mM PMSF and 1 x cOmplete protease inhibitor tablet per 50 ml, and sonicated. The lysate was cleared by centrifugation at 58 000 g for 50 min at 6°C in a JA25 : 50 rotor. The supernatant was incubated with Ni-NTA beads (Thermo) for 1.5 h at 4 C. Beads were washed with 40 column volumes of K560 lysis buffer and proteins were eluted with 250 mM imidazole. Elution fractions were concentrated and loaded onto a Superose 6 Increase 10/300 column (GE Healthcare) pre-equilibrated with K560 gel filtration buffer (50 mM Tris pH 7.5, 300 mM KCl, 1 mM MgCl_2_, 1 mM EGTA, 0.1 mM ATP and 1 mM DTT). Fractions containing K560-2mNeon were snap frozen with 10% glycerol and stored at −70°C.

### 4.2. Total internal reflection fluorescence microscopy

Microscopy was performed on a Zeiss Axio Observer Z1 TIRF microscope using a 100 × NA 1.46 objective equipped with a Photometrics Evolved Delta EMCCD camera and controlled by Zen Blue 2.3 software. For single-molecule experiments, a 1.6 x tube lens was used. The environmental chamber was incubated at 30°C for all experiments. Coverslips used for motility assays were silanized as in [32]. Flow chambers were prepared by attaching a silanized coverslip to a microscopy slide with double-sided sticky tape. Four sample flow chambers were constructed per microscopy slide, each with a volume of 7–8 µl. Rhodamine microtubules were captured using a 561 nm laser with 15% intensity, 75 ms exposure. Images of mNeonGreen and GFP tagged motors were captured using a 488 nm laser with 50% intensity, 100 ms exposure and a frame rate of 0.12 frames per second.

For all *in vitro* motility experiments, 0.2 mg ml^−1^ GMPCPP (Jena Biosciences) microtubule seeds containing 7% rhodamine-tubulin (Cytoskeleton Inc., TL590M-B) were polymerized in BRB80 (80 mM PIPES pH 6.9, 1 mM EGTA and 1 mM MgCl_2_) for 1 h at 37°C, followed by centrifugation at 13 300 rpm for 10 min and then resuspended in BRB80. For gliding assays, anti-His tag antibodies (Raybiotech, 168-10481) at a 1 : 10 dilution in BRB80 were first introduced to the chamber. Next, 40 µl of 1% Pluronic F-127 (Sigma Aldrich) in BRB80 was washed through the chamber and incubated for 5 min. Two hundred nanomolar of purified kinesin motors were then added to the chamber in BRB80 supplemented with 2 mM ATP. Chambers were then washed with 1 mg ml^−1^ casein (Sigma Aldrich) before a 1 : 25 dilution of GMPCPP microtubules was added in the final assay mix (80 mM PIPES pH 6.9, 5 mM MgCl_2_, 2 mM ATP, 1 mM DTT and an oxygen scavenger mix: 0.2 mg ml^−1^ glucose oxidase, 0.035 mg ml^−1^ catalase, 4.5 mg ml^−1^ glucose and 0.1% beta-mercaptoethanol).

For single-molecule motility assays, anti-β-tubulin antibodies (Sigma-Aldrich, T718) at a 1 : 10 dilution in BRB80 were first introduced to the chamber. Next, 40 µl of 1% Pluronic F-127 in BRB80 was washed through the chamber and incubated for 5 min. GMPCPP microtubules were diluted 1 : 50 in BRB80 and then added to the chamber for 5 min. Chambers were then washed with 1 mg ml^−1^ casein (Sigma Aldrich). Freshly purified motors were then added in the final assay mix at concentrations indicated in the figure legends and chambers sealed with nail varnish. For photobleaching and intensity analysis, 0.5 nM of fluorescently tagged motor was added to silanized coverslips and allowed to non-specifically adsorb to the surface. After 3 min, BRB80 supplemented with oxygen scavenger mix was flown through the chamber to wash away non-adsorbed motors. The sample chamber was imaged using the same conditions as described for single-molecule assays.

### 4.3. Image processing and analysis

Kymographs were manually generated in ImageJ [52]. Gliding velocities, single-molecule velocities, run lengths and residency times were measured from these kymographs. Data are collected from at least two independent experiments using motors from separate protein purifications. Histograms were generated from raw velocity data and fit to a Gaussian distribution in MATLAB (Mathworks). Velocities are reported as the mean of the Gaussian fit ± s.e. of the mean. Velocities were calculated as an average of the whole run including pauses. Landing events of less than five frames were not included in analysis. For run lengths and residency times determination, cumulative frequency distributions were generated using the ecdf function in MATLAB and fitted to a single-exponential distribution. Run length and residency times were represented by the decay constant. Landing rates were determined at a concentration of 3.5 nM for each dimeric motor. Welch's *t*-tests were carried out in Graphpad Prism (GraphPad Software). Aggregates as determined from their initial intensity were excluded from analysis. Where necessary, images were also corrected for stage drift using the ImageJ Manual Drift Correction plug-in.

A custom ImageJ macro was developed for analysis of photobleaching steps (https://github.com/bcraske/ImageJ). Briefly, individual fluorescent spots adsorbed to coverslips were manually selected using the multi-point tool in ImageJ. Next, a 4 × 4 pixel square was assigned to the ROI and the average intensity was measured over time using the plot *z*-axis profile function. Background fluorescence was subtracted by assigning a 10 × 10 square centred around the ROI, excluding the 4 × 4 pixel area, using the plot *z*-axis profile function. Discrete photobleaching steps were manually counted from the plotted results. Initial intensity values were calculated as the average fluorescence intensity from a single-adsorbed motor over the first five frames of imaging and the data was plotted as a histogram in MATLAB.

### 4.4. Colabfold analysis

Human CENP-E amino acid sequence 1–483 was analysed using ColabFold: AlphaFold2 using MMseqs2 [25,26]. The complex was modelled using ColabFold by entering the two identical chains of residues 1-483 which were separated by ‘:’ to indicate homodimerization.

## Data Availability

This article has no additional data.
